# A Methodology to Validate the InSAR Derived Displacement Field of the September 7^th^, 1999 Athens Earthquake Using Terrestrial Surveying. Improvement of the Assessed Deformation Field by Interferometric Stacking

**DOI:** 10.3390/s8074119

**Published:** 2008-07-10

**Authors:** Ioannis Kotsis, Charalabos Kontoes, Dimitrios Paradissis, Spyros Karamitsos, Panagiotis Elias, Ioannis Papoutsis

**Affiliations:** 1 Higher Geodesy Laboratory and Dionyssos Satellite Observatory, National Technical University of Athens, Iroon Polytexneiou 9, 15780, Zografou, Greece; E-Mails: jkotsis@survey.ntua.gr (I.K.); dempar@central.ntua.gr (D.P.); karamits@central.ntua.gr (S.K.); 2 National Observatory of Athens, Institute of Space Applications and Remote Sensing, Vas. Pavlou and Metaxa str., 15236, Palaia Penteli, Greece; E-mails: pelias@space.noa.gr (P.E.); ipapoutsis@space.noa.gr (I.P.)

**Keywords:** Ground subsidence, SAR Interferometry, stacked interferogram, leveling

## Abstract

The primary objective of this paper is the evaluation of the InSAR derived displacement field caused by the 07/09/1999 Athens earthquake, using as reference an external data source provided by terrestrial surveying along the Mornos river open aqueduct. To accomplish this, a processing chain to render comparable the leveling measurements and the interferometric derived measurements has been developed. The distinct steps proposed include a solution for reducing the orbital and atmospheric interferometric fringes and an innovative method to compute the actual InSAR estimated vertical ground subsidence, for direct comparison with the leveling data. Results indicate that the modeled deformation derived from a series of stacked interferograms, falls entirely within the confidence interval assessed for the terrestrial surveying data.

## 1. Introduction

One of the most significant natural disasters to strike Greece in the 20^th^ century was the September 7, 1999, 11^h^ 56^m^ 50^s^ UTC, Mw (moment magnitude) = 5.9 Athens earthquake. It claimed the lives of 143 people, and caused the collapse of several buildings, mainly in the northwest suburbs of the Greek capital. The approximate location of the earthquake epicenter was 38.10°N, 23.56°E, roughly 20 km northwest from the center of Athens [1].

The vertical displacement field at the surface level caused by this tectonic event was investigated with space born Synthetic Aperture Radar Interferometry (InSAR), using ERS-2 data. InSAR processing showed a significant deformation with the maximum Line Of Sight (LOS) subsidence being of approximately 6 cm [1]. This observation was used in earthquake modeling and fault location mapping [2-9] along the middle of the Parnitha mountain. However, the deformation field reported in [1] could not be verified at that time due to the lack of co-seismic geodetic measurements of adequate precision. The sole indication was provided by geologists and engineers who visited the area and confirmed that the damaged structures, at the substructure level, were showing a vertical movement of the same order of magnitude as the InSAR derived assessments.

**Figure 1.**
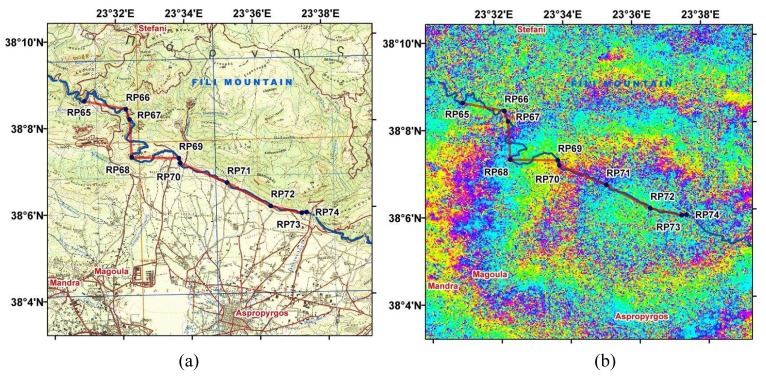
(a) Plots of the Mornos aqueduct (blue) and height network (red) projected on 1:50000-scale map and (b) onto an ERS-2 SAR image interferogram.

The region of maximum deformation coincided with the main shock epicenter. This area was very close to the Mornos river open aqueduct, used for water supply to Athens. The distance of the aqueduct pass from the earthquake epicenter was less than 2.5 km. The water supply authority in Athens awarded an aqueduct-leveling project to the National Technical University of Athens/Higher Geodesy department (NTUA/HG), which lasted for two months, from March to April 2001. Prior leveling data along the Mornos aqueduct had been obtained in 1984. No height data were available for the intermediate time interval 1984-2001; however no major seismic event had taken place in that period. The two co-seismic sets of leveling data were considered adequate to investigate the vertical displacement in the affected by the earthquake area and verify the InSAR derived observations. Figure 1(a) illustrates the leveling path legs and the Mornos aqueduct projected onto the 1:50,000-scale map. Figure 1(b) shows the area where leveling data were acquired, projected onto the calculated interferogram. The test area extends from 38°09′N 23°31′E to 38°06′N 23°38′E.

The scope of this paper is the evaluation of the InSAR derived displacement field caused by the Sept 7, 1999 Athens earthquake, using as reference an external data source provided by terrestrial surveying along the Mornos river open aqueduct. Research works relating to InSAR – leveling interoperability issues have been published in the past, focusing on either verifying the InSAR derived subsidence, or integrating them with the leveling data to increase the reliability of the measurement. In [10] a spatially dense network of leveling benchmarks was used, to integrate terrestrial measurements with InSAR data, and sums of Gaussian surfaces were proposed to approximate the subsidence field induced from oil/gas extraction activity. Moreover, in [11] a method to improve the InSAR derived deformation field was presented, by splitting the differences between InSAR and leveling derived assessments to two components: one mathematical model accounted for the mean tropospheric effects and orbital errors, and a second model was used to describe for the local, less correlated error sources, such as Digital Elevation Model (DEM) errors and local atmospheric effects. By approximating models with polynomials and by generating a non – mathematical model for the residuals of the approximations, corrections for the InSAR derived deformations were produced for the entire SAR image. In [12] a study for mine subsidence monitoring using ERS-1/2 and JERS-1/2 was investigated, combining the resulted subsidence with ground-collected data. In [13] InSAR derived deformations were compared and correlated with temporally dense leveling data for settlements monitoring in the reclaimed land of the new Hong Kong international airport and the Fairview Park.

This paper is structured as follows: section 2 refers to the preliminary processing of the input data, namely the InSAR and leveling measurements. Section 3 presents in an analytic way the distinct steps in rendering the two data sets compatible. Section 4 outlines the results obtained by applying the proposed processing chain, whilst section 5 investigates more thoroughly the physical meaning of these results and the applicability of the method in verifying InSAR derived subsidence on the basis of terrestrial surveying data.

## 2. Input Data

### 2.1. ERS-1/2 InSAR Data

ERS-1/2 sensor images spanning the period from December 1997 to January 2001 were acquired and processed over the Athens Greater Area. The satellite images were provided by the European Space Agency in the frame of the ESA-GREECE AO project 1489OD/11-2003/72.

**Figure 2.**
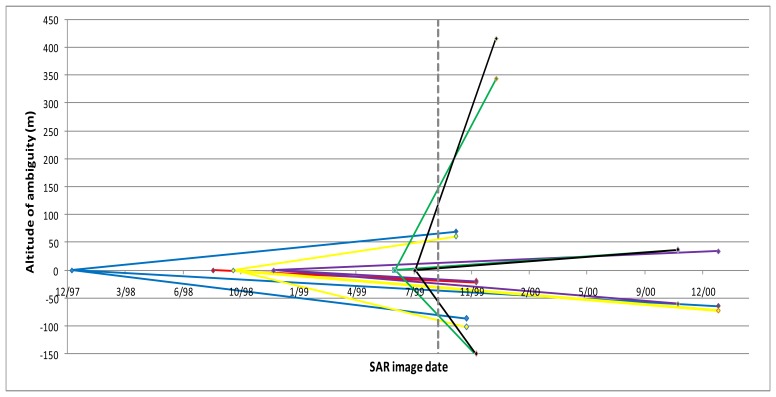
Set of interferometric pairs used in the study. The vertical dashed line indicates the date of the earthquake occurrence.

Interferometric calculations were done by using the CNES DIAPASON InSAR processing software, and the sixteen most coherent co-seismic interferograms were kept for the purposes of the study. The image pairs used along with their corresponding “altitude of ambiguities” are shown in Figure 2. The influence of the terrain relief on the interferograms was lifted out using a DEM, which was originated by digitizing the 20 m contour lines from the 1:50,000-scale topographic maps. The high frequency DEM artifacts remaining in the interferograms, were calculated as the ratio of the DEM error (∼10 m) over the interferometric “altitude of ambiguity” (20 m–417 m) [14]. They were all estimated to be below the cycle level (0.3–0.02 cycles).

### 2.2. Leveling Data Along the Mornos Aqueduct

The first terrestrial surveying work on the aqueduct was done in 1984, covering its whole length of approximately 200 km. A special trigonometric height technique was used, providing the same level of accuracy as conventional leveling but being significantly faster [15]. This technique employed a highly accurate geodetic total station to obtain the slope distance and the vertical angle between the two points of interest. The use of a redundant number of stationary sets of tripods and tribranch adapters eliminated the need for target and instrument height measurements. Furthermore, atmospheric refraction effects were further eliminated by concurrent measurements at both ends of an observation line - leading to high accuracy observations.

**Figure 3.**
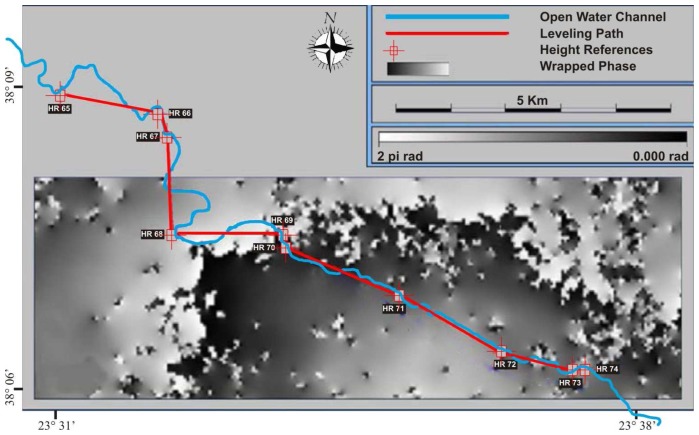
Leveling path legs plot (red) and aqueduct plot (blue) projected onto a wrapped interferogram. For clarity purposes, only the segments connecting the height references are displayed. The actual leveling path follows the channel.

Moreover, a standard geometric leveling was realized in 2001. The total distance surveyed was 40 km, of which 12 km were confined in the area of interest illustrated in Figure 1(b). Figure 3 shows the leveling path legs and the longitudinal axis of the open aqueduct, projected onto a wrapped interferogram.

The accuracy of the leveling works was estimated to be of the order of a few millimeters between successive height references [16]. It should be noted that the two leveling experiments conducted in years 1984 and 2001 used exactly the same height reference points. The height differences obtained by surveying the aqueduct at the two epochs indicated a significant vertical displacement induced by the earthquake. Taking into account the standard deviations of the geometric leveling and the trigonometric leveling and by applying the error propagation law, the standard deviations of the height differences were estimated to range from 4 mm to 8 mm. These values correspond to the relative heights between successive height benchmarks, depending on the length of the leveling path segments.

## 3. Rendering InSAR Data Comparable to Leveling Data

The differential displacement data derived by the two different techniques were incompatible and consequently a direct comparison was not possible. These incompatibilities may be summarized as follows:

- InSAR processing provided wrapped interferograms, consequently only the fractional part mod_2π_Φ of the full phase difference Φ was known.
- InSAR results correspond to the projection of the true vertical deformation along the LOS vector.
- The reference systems of the leveling data and the InSAR data were different. InSAR data were referring to ED 50 UTM zone 34 while the leveling data were referring to the mean sea level and the height reference positions to the Hellenic Geodetic Reference System 87 (HGRS 87).
- The interferograms were “noisy” mainly due to temporal decorrelation, orbital and tropospheric disturbances.

The following sections describe the procedure used to eliminate the effects of the above types of incompatibility, rendering the two datasets comparable.

### 3.1. Wrapped Interferogram Filtering

**Figure 4.**
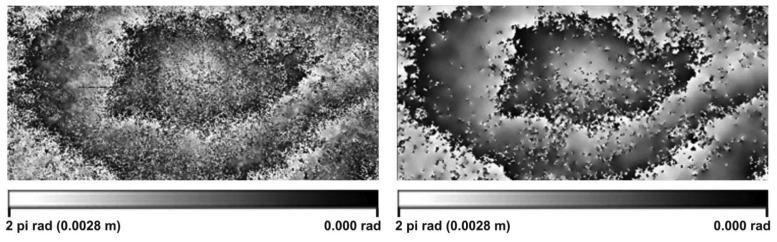
Wrapped interferogram, before (left) and after (right) filtering.

The wrapped interferogram underwent a simple filtering procedure. The primary objective of this action was to minimize the probability of phase unwrapping failure, while a secondary goal was the improvement of the wrapped and unwrapped interferogram appearance in order to derive qualitative evaluations more efficiently. The filter used was a simple 2D 3×3 space mean filter (symmetric to match the rectangular pixel dimensions), applied on both the real cos(ψ_i,j_) and imaginary sin(ψ_i,j_) parts of a virtual unitary magnitude signal e^jψi,j^ = cos(ψ_i,j_) + j sin(ψ_i,j_) . The phase of this signal is the unfiltered interferometric phase ψ_i,j_ In other words, the 2D space filter was applied on a unitary signal to which the phase of the input interferogram was projected. The phase ψ_flt/i,j_, comprising the filtered interferogram, was extracted through an arctan operation from the filtered real and imaginary parts of the virtual signal. The filtering procedure is best defined by the following formula:

(1) $$\Psi_{\text{flt}/\text{i},\text{j}}=\text{arctan}\left(\sum_{\text{j}={\text{j}}_{0}-\frac{\text{k}-1}{2}}^{\text{j}={\text{j}}_{0}+\frac{\text{k}-1}{2}}\sum_{\text{i}={\text{i}}_{0}-\frac{k-1}{2}}^{\text{i}={\text{i}}_{0}+\frac{\text{k}-1}{2}}\frac{\text{sin}\left(\psi_{\text{i},\text{j}}\right)}{{\text{k}}^{2}}/\sum_{\text{j}={\text{j}}_{0}-\frac{\text{k}-1}{2}}^{\text{j}={\text{j}}_{0}+\frac{\text{k}-1}{2}}\sum_{\text{i}={\text{i}}_{0}-\frac{\text{k}-1}{2}}^{\text{i}={\text{i}}_{0}+\frac{\text{k}-1}{2}}\frac{\text{cos}\left(\psi_{\text{i},\text{j}}\right)}{{\text{k}}^{2}}\right)$$

where k is the filter size, which equals 3. This value was considered to be an optimal one, as it corresponds to a satisfactory tradeoff between interferometric spatial resolution and level of smoothing. The criterion for choosing k was to eliminate isolated pixel noise while keeping the spatial deformation trend evident in the interferogram. In Figure 4, the effect of the interferogram filtering procedure is presented.

### 3.2. Phase Unwrapping

Various 2D phase unwrapping techniques have been developed for resolving the “integer ambiguity” problem of the interferometric phases. In this study “Quality Guided Path Following”, “Least Squares Without Weights”, “Weighted Least Squares”, and “Minimum LP Norm” approaches were implemented [17-20]. The unwrapped interferograms produced by these techniques were evaluated for surface discontinuities, by inspecting for the presence of breaklines (abrupt gradient changes) or “tears” (non – derivabilities) and measuring their length. As a result, it was inferred that the most effective technique, for this particular scenario, was the “Weighted Least Squares”. The weights were derived from the coherence map, representing the computed cross correlation between the master and the slave image.

**Figure 5.**
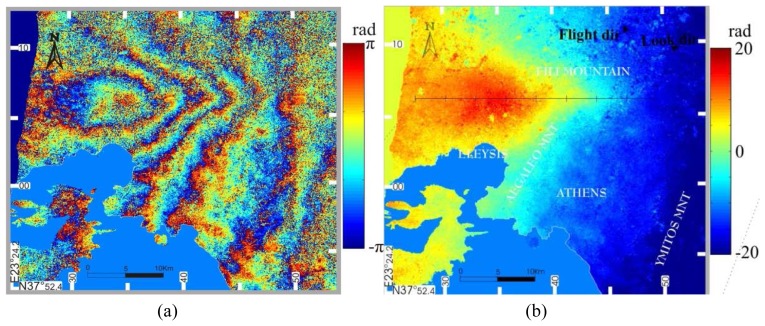

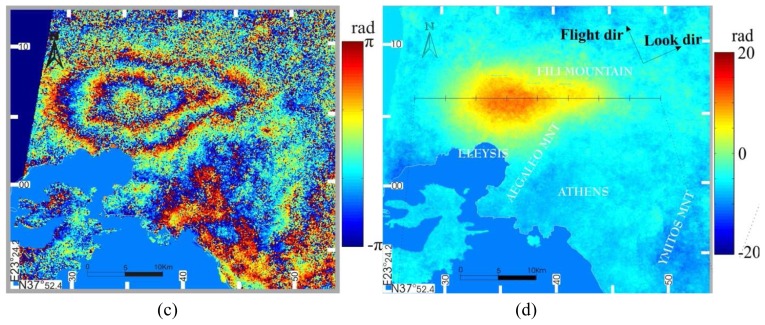
(a) & (b) Wrapped and unwrapped versions of the same interferogram. Note the unrealistic fringe pattern due to inaccuracies in the orbital data used. (c) & (d) The effect of the “tilting” and “shifting” operation on the same interferogram; the orbital fringes are removed.

The unwrapped co-seismic interferograms were all undergone a special processing in order to minimize the existing orbital, tropospheric and DEM disturbances. These errors were lifted by a “tilting” and “shifting” operation, using a number of coherent pixels located outside the deformed area. According to this approach [21], the deformation on these pixels was expected to follow a well-defined t-student distribution around a local zero mean. Then, by forcing each local deformation mean to zero, the calculated interferograms were “tilted” and “sifted”. Figure 5 emphasizes the effect of this process, where the disposal of the orbital fringes becomes evident.

### 3.3. Incidence Angle Correction

**Figure 6.**
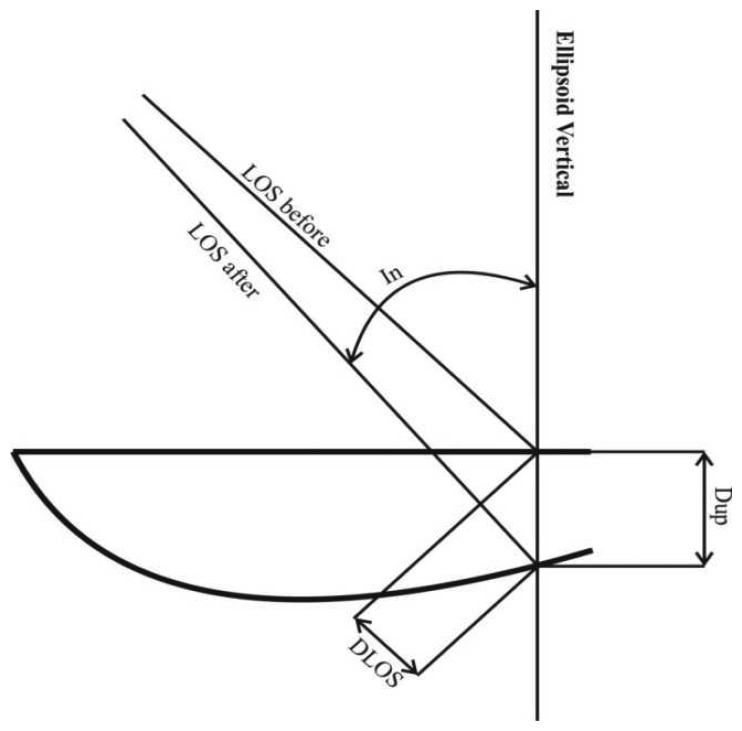
Relative geometry of the true vertical deformation and the deformation provided by InSAR.

The HGRS 87 unwrapped interferogram provides the differential vertical displacements for each target pixel as projected to the LOS vector **Φ**_LOS_(E,N) , and not the vertical differential displacements**Φ**_dU_(E,N) themselves, as is the case of leveling (Figure 6). These two quantities are related through the incidence angle In(E, N):

(2) $$\Phi_{\text{LOS}}(\text{E},\text{N})=\Phi_{\text{dU}}(\text{E},\text{N})\cdot \cos(\text{In}(\text{E},\text{N}))$$

In order to determine the differential vertical displacements from LOS projection displacements, the value of the incidence angle for each target pixel was required. The incidence angle computation procedure was based on satellite trajectory data and the position of the target. Initially, for every target pixel (i, j) the zero - Doppler position of the space born SAR sensor had to be computed. This was achieved through signal processing applied on the “master” (or “reference”) image. Third degree polynomials were fitted with Least Squares to the known satellite position vectors **r**(t) derived by ERS 1/2 operational orbits provided in the header file of every SAR image. These expressions simply provide the satellite position vectors in the orbit's terrestrial geocentric reference frame as a function of time. Three polynomials were derived, one for every coordinate X, Y and Z. Exactly the same procedure was applied for the satellite velocity vector **ṙ**(t) and three additional equations were also obtained. Therefore, for every single target (i, j) the following procedure was followed:

1. The map projection coordinates of the target were converted to geocentric Cartesian coordinates in the geodetic terrestrial reference frame in which the satellite orbits were provided (in this particular case from HGRS 87 map coordinates to ITRF 96 geocentric Cartesian coordinates).
2. The mean Doppler frequency shift was computed by the CNES DIAPASON software and was assumed to be the same for every single pixel target. The Doppler frequency shift f(i,j) was expressed as a function of the satellite position, the satellite velocity vectors and the target position **r**(i, j), by the following equation (λ denotes the SAR sensor wavelength):
  (3) $$\text{f}(\text{i},\text{j})=\frac{2(\text{r}(\text{i},\text{j})-\text{r}({\text{t}}_{\text{i}}))\cdot \dot{\text{r}}(\text{i},\text{j})}{\lambda |\text{r}(\text{i},\text{j})-\text{r}({\text{t}}_{\text{i}})|}$$
3. A total of seven equations were accumulated, and an equal number of unknowns was introduced, three for the satellite position vector, three for the satellite velocity vector and one for the time t_i_. Hence, a non linear seven-equation system was created for the estimation of the seven unknowns. The system was linearised with Taylor series expansion and solved iteratively.
4. Knowing the satellite and target position vectors, the unitary LOS vector could be calculated simply from the following vector equation:
  (4) $$\text{LOS}\left(\text{i},\text{j}\right)=\frac{\text{r}\left(\text{i},\text{j}\right)-\text{r}{(\text{t}}_{\text{i}})}{|\text{r}\left(\text{i},\text{j}\right)-\text{r}{(\text{t}}_{\text{i}})|}$$
5. The target position ellipsoidal coordinates φ_i,j_, λ _i,j_ were then calculated on the same geodetic terrestrial frame, which was used to express the orbits and the target coordinates in the previous step.
6. Knowing the target's latitude and longitude φ_i,j_, λ _i,j_ the LOS vector components were transformed to the local geodetic reference system (delta north - DN, delta east - DE, delta up -DU) by means of a rotation matrix:
  (5) $$[\begin{array}{l} \begin{array}{c} \text{DX}(\text{t})\\ \text{DY}(\text{t})\\ \text{DZ}(\text{t}) \end{array}] \end{array}\text{R}\left(\varphi_{\text{i},\text{j}}{,\lambda }_{\text{i},\text{j}}\right)=\left[\begin{array}{l} \text{DN}(\text{t})\\ \text{DE}(\text{t})\\ \text{DU}(\text{t}) \end{array}\right]$$
7. The third component of the **LOS** vector as expressed in the local geodetic reference system is actually the direction cosine for the “up” axis of the system, and consequently the cosine of the incidence angle In. Thus the incidence angle can be derived as:
  (6) In=arctan(DU)

#### 3.4. Stacking

**Figure 7.**
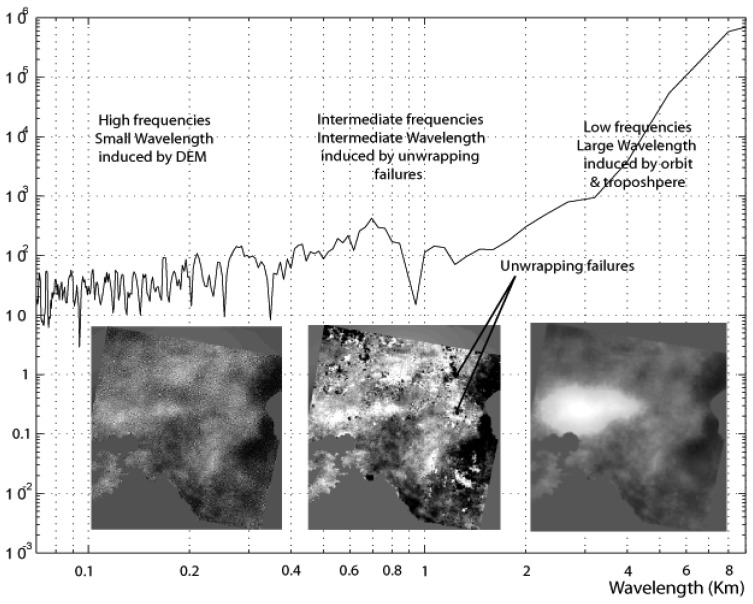
Spectral density of the stacked interferogram. Low frequencies prevail.

In the framework of this study and due to the fact that reliable verification data were available through the leveling survey, it was possible to evaluate the advantage in using a mean stacked interferogram instead of using only one, that is the “highest-quality” (most coherent) interferogram. For this purpose the sixteen “tilted” and “shifted” unwrapped interferograms were stacked to derive a mean temporal deformation field. This technique produced an image S(i, j) defined as: S(i, j) = mean(I_1_(i, j), I_2_ (i, j),… I_n_ (i, j)), where n represents the number of the available interferograms and I_m_(i, j) the unwrapped interferometric phase of the m^th^ interferogram at pixel location (i,j). Consequently the produced interferogram depicting the mean deformation field, was released from high and intermediate frequencies [21], which corresponded to non-earthquake related interferometric disturbances (Figure 7).

It should be mentioned that at this stage alternative stacking methods were implemented as well. They comprised of the formation of A) a weighted mean stacked interferogram, using as weights the pixel coherence values of each contributing interferogram, B) a maximum coherence stacked product, on which each phase pixel value stems from the interferogram with the highest corresponding coherence pixel value and C) a windowed maximum coherence stacked product; here each phase pixel value stems from the interferogram with the highest mean coherence value, calculated inside a 3 by 3 pixels window, centered on the pixel of interest. As is shown in section 4, the above methods returned very similar results compared to the mean stacked approach.

### 3.5. Geodetic Reference System Conversion

As mentioned the unwrapped interferometric calculations were referring to a UTM map projection on the ED 50 Greek Datum. In contrast the coordinates of the height references were expressed in the HGRS 87 reference system, using the Transverse Mercator map projection on the GRS 80 ellipsoid. To overcome this incompatibility the initial interferograms were converted to HGRS 87 projection system as follows:

1. The ED 50 UTM map coordinates (Eastings and Northings - E, N) were converted to ED 50 ellipsoidal coordinates (latitude and longitude -φ, λ), assigning to each pixel the corresponding orthometric height (H) derived from the input DEM.
2. The orthometric heights were converted to geometric ones (h), by implementing a constant additive geoid undulation value (N) for the entire area of interest, since the geoid in this area is relatively “flat” exhibiting a very low gradient. This value was obtained by the Ohio State University OSU 91 Geoid Model, and was recomputed for ED 50.
3. The ED 50 ellipsoidal coordinates were converted to ED 50 Cartesian coordinates (X, Y, Z).
4. Subsequently, the ED 50 geocentric Cartesian coordinates were converted to HGRS 87 geocentric ones, assuming only a parallel shift between the two systems. The latter assumption was expected to successfully provide the conversion due to the small size of the area of interest.
5. Then, the HGRS 87 geocentric Cartesian coordinates were translated to HGRS 87 ellipsoidal (φ, λ) coordinates.
6. Ultimately, the HGRS 87 ellipsoidal (φ, λ) coordinates were converted to HGRS 87 Transverse Mercator projection coordinates (E, N).

### 3.6. Differential Vertical Displacement Modeling

Thorough examination of the unwrapped (stacked and/or “highest-quality”) interferograms, exhibited the presence of “local” phase anomalies in certain areas extending from one to several pixels. The phase values in these pixels deviated from the prevailing values in the surrounding region. These anomalies were survived the filtering procedure described in section 3.1. It is beyond the scope of this paper to explore the origin of such phase “residuals”, but it could be assumed that they stemmed from local temporal decorrelation. It was also observed that the areas affected by these anomalies, presented significantly low coherence values and therefore they should be excluded.

Because the tectonic deformations observed were characterized by low phase gradient and spatial continuity, it was decided to proceed with a phase smoothing operation, by fitting (with the Weighted Least Squares method) a 3D-mathematical surface to the unwrapped interferometric phases **Φ**_dU_(E, N). After a series of adjustments, a successful fit according to the chi-square (*χ*^2^) test was achieved, using the value of 6 mm as a-priori standard deviation for the observations. By the application of the error propagation law (given the estimated model parameters and their a-posteriori standard deviation values), it was concluded that the 3D-mathematical surface would provide the vertical deformation estimate for each target pixel (E, N), with an estimated a-priori deviation not higher than 0.2 mm. In order to ensure that the mathematical model represents the best fit to the displacement pattern observed, the most general form of m^th^ degree surface was tested:

(7) $$\Phi_{\text{dU}}\left(\text{E},\text{N}\right)=-\left(\begin{array}{l} {\text{a}}_{0}{+\text{a}}_{{\text{E}}_{1}}{\text{E}}^{1}{+\text{a}}_{{\text{E}}_{2}}{\text{E}}^{2}{+\ldots \text{a}}_{{\text{E}}_{\text{m}}}{\text{E}}^{\text{m}}+\\ {\text{a}}_{{\text{N}}_{1}}{\text{N}}^{1}{+\text{a}}_{{\text{N}}_{2}}{\text{N}}^{2}{+\ldots \text{a}}_{{\text{N}}_{\text{m}}}{\text{N}}^{\text{m}}{+\text{a}}_{\text{EN}}{\text{E}}^{1}{\text{N}}^{1}+\\ {\text{a}}_{{\text{E}}_{1}{\text{N}}_{\left(\text{m}-1\right)}}{\text{E}}^{1}{\text{N}}^{\left(\text{m}-1\right)}{+\text{a}}_{{\text{E}}_{2}{\text{N}}_{\left(\text{m}-2\right)}}{\text{E}}^{2}{\text{N}}^{\left(\text{m}-2\right)}{+\ldots \text{a}}_{{\text{E}}_{\left(\text{m}-1\right)}{\text{N}}_{1}}{\text{E}}^{\left(\text{m}-1\right)}{\text{N}}^{1} \end{array}\right)$$

**Figure 8.**
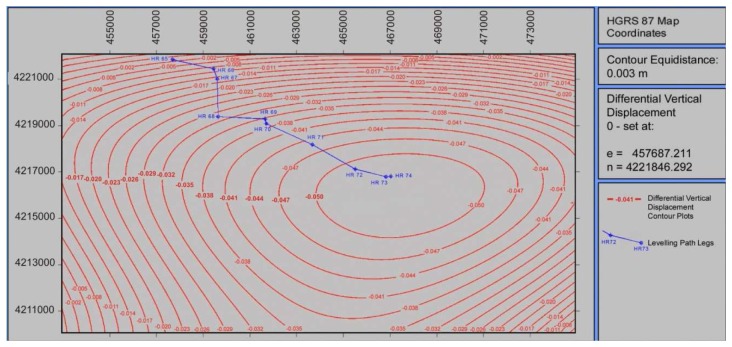
Differential vertical displacement model using a third degree mathematical surface.

After several runs, it was determined that a polynomial surface with degree higher than third would be redundant, as it was not offering any further improvement in terms of a-posteriori variance and measurement residuals. All higher degree coefficients were close to zero. The produced surface is presented in Figure 8. A Gaussian 3D surface was also tested; however this model was far less successful, mainly due to the absence of axial symmetry of the deformation pattern.

## 4. Results

**Figure 9.**
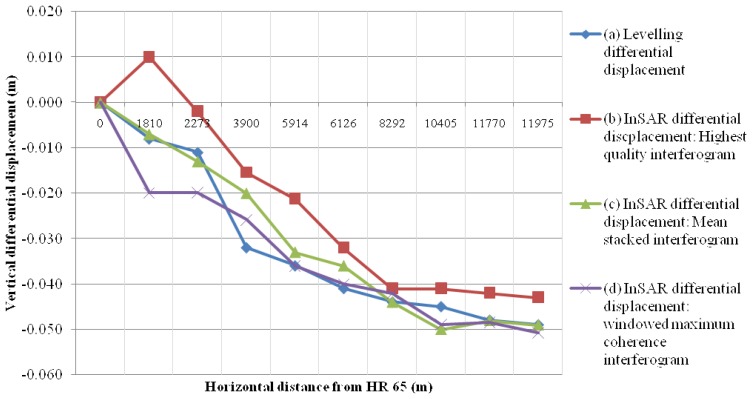
Differential vertical deformation profiles derived by the, (a) conventional terrestrial surveying, (b) single “highest quality” interferogram, (c) mean stacked interferogram, (d) windowed maximum coherence interferogram. HR65 indicates the starting point of leveling.

Based on the 3D surface model produced, it became possible to extract a profile section of the InSAR vertical differential displacements along the leveling traverse. For this an origin had to be defined, and this was decided to be the height reference HR 65. Consequently, its displacement was set to zero. All other vertical displacements were provided in relevance to HR65. Profile data for InSAR and leveling data are presented in Figure 9.

Examining the profiles illustrated in Figure 9, it can be concluded that no major differences occur between the differential vertical displacements as obtained by InSAR and leveling. There appears to be an agreement between the two profiles with respect to the gradient of the vertical displacement. Also there is no evidence of any systematic deviation between them. Moreover the profile corresponding to the mean stacked interferogram shows a better agreement with the leveling data. The vertical displacement differences between the leveling data and the interferometric data using the “highest quality” interferogram range from 3 mm up to 1.8 cm. The average difference value between the two data sets is 9.5 mm and the standard deviation equals 5.5 mm. On the contrary, when the mean stacked interferogram is compared with the leveling data, the above discrepancies are reduced by a factor of six. Indeed, the average difference between the two data sets is reduced down to 1.5 mm, whereas the standard deviation is of the order of 4.8 mm.

Table 1 outlines the average vertical displacement difference between the leveling data and the interferometric data for the various interferometric approaches used. The study of the table shows that the mean stacked product is preferred against the other interferograms, as it fits precisely the leveling data. Also its estimation entails less computational complexity. It should be mentioned though, that there are no major differences between the various stacking methods. However, significant improvement was achieved when moving from the single most coherent interferogram to any of the stacked products.

## 5. Conclusions – Discussion

This research focused on rendering compatible and comparable the InSAR derived displacements, related to the September 7, 1999 Athens earthquake, with leveling survey data. Towards this goal a processing chain was implemented, encompassing an algorithm for orbit and atmospheric disturbances removal, and a methodology for transforming the LOS deformation vector to the true vertical deformation vector. The proposed method used a mean stacked interferogram to get a more consistent representation of the displacement pattern. Finally, an agreement between the deformation values originating from InSAR data with the ones derived from leveling survey data was demonstrated. Only minor discrepancies were identified between the two.

These small differences may be attributed to several types of error sources, such as 1) SAR sensor noise, radiometric instabilities and system aging, 2) surface subsidence model deviations, 3) remaining orbital phase “ramps”, 4) remaining tropospheric artifacts, 5) unwrapping errors, 6) temporal decorrelation effects, and 7) DEM errors. The possibility for a-seismic deformations in the period 1984-1998 could be also considered as a possible contributor to the relative subsidence profile differences. However, this a-seismic tectonic deformation, if it exists, remains unaccounted for, due to the absence of InSAR calculations in that period. The above-mentioned factors, may contribute to the observed total error of the derived relative subsidence values. However, the combined influence of the first three factors is considered to be essentially ignorable, taking into account the orders of magnitude of the resulting relative vertical displacement differences. Moreover, unwrapping errors computed by rewrapping the unwrapped interferogram, were assessed to be to an acceptable level in the area of interest. Therefore factors 6 and 7 namely temporal decorrelation and DEM errors, seem to be the most crucial parameters resulting in InSAR subsidence profile deviations. Temporal decorrelation could not be computed but must be considered as the major contributor to the spatially uncorrelated component of the residuals arising from the Least Squares approximation of the polynomial surface.

However as shown in Figure 9, the influence of all disturbing factors described previously, was effectively reduced by using a mean stacked and noise-free interferogram. Moreover the suggested tilting and shifting procedure, introduced in [21], for removing orbital and tropospheric fringes has performed effectively. Hence, the earthquake induced subsidence pattern seemed to be successfully represented by the proposed model.

As far as the terrestrial surveying derived relative subsidence profiles are concerned, the estimation accuracy was much simpler and more explicit. The leveling data accuracy was estimated to lie in the range from 4 mm to 8 mm, in relative heights between successive height benchmarks. With the above estimations it becomes clear that the deviation of the two relative subsidence profiles (cases (a) and (c) in Figure 9), fall entirely within the confidence interval defined for the leveling data. It can be also concluded that the simple polynomial surface modeling of the subsidence field, may be regarded as an effective method to overcome the remaining temporal decorrelation effects and other sources of noise, by exploiting the extremely high degrees of freedom associated with the Least Squares approximation of mathematical models. Finally, a case specific conclusion of geophysical interest can be drawn for the study area. This refers to the fact that no detectable significant vertical displacements have occurred during the period 1984-1998, for which InSAR interferometric measurements were not available.

## Figures and Tables

**Table 1.** Average and standard deviation of the vertical displacement differences between the leveling data and the InSAR methods.

	**Highest quality interferogram**	**Mean stacked interferogram**	**Weighted mean stacked interferogram**	**Maximum coherence stacked interferogram**	**Windowed maximum coherence stacked interferogram**
**Average difference (m)**	−0,0096	−0,0016	−0,0030	0,0047	0,0020
**Standard deviation (m)**	0,0056	0,0048	0,0055	0,0150	0,0056

## References

[1] Kontoes C., Elias P., Sykioti O., Briole P., Remy D., Sachpazi M., Veis G., Kotsis I. (2000). Displacement Field Mapping and Fault Modeling of the Mw = 5.9, September 7, 1999 Athens Earthquake based on ERS-2 Satellite RADAR Interferometry. Geophysical Research Letters.

[2] Roumelioti Z, Dreger D., Kiratzi A., Theodoulidis N. (2003). Slip distribution of the 7 September 1999 Athens earthquake inferred from an empirical Green's function study. Bulletin of the Seismological Society of America.

[3] Baumont D., Courboulex F., Scotti O., Melis N.S., Stavrakakis G. (2002). Slip distribution of the M-w 5.9, 1999 Athens earthquake inverted from regional seismological data. Geophysical Research Leters.

[4] Papadimitriou P., Voulgaris N., Kassaras I., Kaviris G., Delibasis N., Makropoulos K. (2002). The M_w_=6.0, 7 September 1999 Athens earthquake. Natural Hazards.

[5] Sargeant S.L., Burton P.W., Douglas A., Evans J.R. (2002). The source mechanism of the Athens earthquake, September 7, 19999, estimated from P seismograms recorded at long range. Natural Hazards.

[6] Pavlides S.B., Papadopoulos G., Ganas A. (2002). The fault that caused the Athens September 1999 Ms=5.9 earthquake: Field observations. Natural Hazards.

[7] Goldsworthy M., Jackson J., Hains J. (2002). The continuity of the active fault systems in Greece. Geophysical Journal International.

[8] Bouckovalas G.D., Kouretzis G.P. (2001). Stiff soil amplification effects in the 7 September 1999 Athens (Greece) earthquake. Soil Dynamics and Earthquake Engineering.

[9] Eftaxias K., Kapiris P., Polygiannakis J., Borgis N., Kopanas J., Antonopoulos G., Peratzakis A., Hadjicontis V. (2001). Signature of pending earthquake from electromagnetic anomalies. Geophysical Research Letters.

[10] Odijk D., Kenselaar F., Hanssen R. Integration of Leveling and InSAR Data For Land Subsidence Monitoring.

[11] Zhou Y., Stein A., Molenaar M. (2003). Integrating Interferometric SAR data with Leveling Measurements of Land Subsidence Using Geostatistics. International Journal of Remote Sensing.

[12] Ge L., Chang H.C., Janssen V., Rizos C. The Integration of GPS, radar Intrerferometry and GIS for Ground Deformation Monitoring.

[13] Liu G., Chen Y., Ding X., Li Z., Li Z.W. (2002). Monitoring Ground Settlement in Hong Kong with Satellite SAR Interferometry. Proc. FIG XXII International Congress Washington 5.

[14] Massonet D., Feigl K. (1998). Radar interferometry and its application to changes in the Earth's surface. Reviews of Geophysics.

[15] Balodimos D. (1979). The development of a special trigonometric leveling technique. Tecnika chronic.

[16] Deltsidis P., Saridakis M. (2001). Displacement determination with terrestrial and satellite methods. Diploma Thesis.

[17] Prit M.D. (1997). Comparison of path-following and least-squares phase unwrapping algorithms. IGARSS.

[18] Zebker H.A., Lu Y. (1998). Phase unwrapping algorithms for radar interferometry: Residue-cut, least squares, and synthesis algorithms. Journal of the Optical Society of America.

[19] Pritt M.D. (1997). Congruence in least-squares phase unwrapping. IGARSS.

[20] Ghiglia D.C., Romero L.A. (1996). Minimum LP-norm two-dimensional phase unwrapping. Journal of the Optical Society of America.

[21] Elias P., Kontoes C., Sykioti O., Avallone A., Briole P., Paradissis D. (2006). A method for minimizing low frequency and unwrapping artefacts from interferometric calculations. International Journal of Remote Sensing.

